# Selection of reliable reference genes during THP-1 monocyte differentiation into macrophages

**DOI:** 10.1186/1471-2199-11-90

**Published:** 2010-12-01

**Authors:** Marten B Maeß, Stefanie Sendelbach, Stefan Lorkowski

**Affiliations:** 1 Institute of Nutrition, Friedrich Schiller University Jena, Dornburger Str. 25, 07743 Jena, Germany

## Abstract

**Background:**

Reliable reference genes are a vital prerequisite for any functional study employing quantitative real-time RT-PCR (RT-qPCR) for analyzing gene expression. Yet a proper selection and assessment of the chosen reference genes is only rarely included into a study. To date, no reference genes have been validated for differentiation of THP-1 monocytes. Here we report on the selection of validated reference genes during differentiation of THP-1 monocytes into macrophages induced by phorbol 12-myristate 13-acetate (PMA).

**Results:**

The mRNA expression of 21 preselected potential reference genes was measured by RT-qPCR at several time-points over six days of PMA-induced THP-1 monocyte-to-macrophage differentiation. A ranking according to expression stability was calculated. Calculations were performed using Microsoft Excel-based applets GeNorm, NormFinder and BestKeeper. Our results indicated ACTB (β-actin) (C_q _± SD, 14.1 ± 0.3) and RPL37A (ribosomal protein L37a) (14.5 ± 0.3) as the most stable genes. While other frequently used reference genes such as GAPDH (glycereraldehyde-3-phosphate dehydrogenase) (20.8 ± 0.8) or G6PD (glucose-6-phophate dehydrogenase) (16.1 ± 1.0) were found to be not as reliable and were therefore unsuited for use as reference genes. These findings were validated by investigating mRNA expression of macrophage scavenger receptor CD36, known to be regulated during monocyte-to-macrophage differentiation. Using ACTB and RPL37A as reference genes a profound and significant regulation of CD36 could be demonstrated, while use of G6PD resulted in a much less pronounced apparent regulation of CD36.

**Conclusion:**

Consequently, it is recommended to normalize any real-time PCR-based expression data obtained during THP-1 monocyte differentiation using ACTB and RPL37A.

## Background

Macrophages are the phagocytic cells of the immune system which play a pivotal role in many disease processes [1]. Upon a local stimulus circulating blood monocytes immigrate into the respective tissue where they differentiate into mature macrophages. Due to the very complex interactions of macrophages with the cells of the surrounding tissue and their manifold activities, which include removal of necrotic and apoptotic tissue or invaded microorganisms, contribution to wound healing, and presentation of antigens [1,2], intensive effort in basic and clinical research has been spend on unraveling the biology of macrophages and their behavior.

Although pure human primary monocytes can be obtained either by dextran sedimentation followed by Ficoll density centrifugation [3], or leukapharesis combined with counter current elutriation [4], the number of primary monocytes for functional studies is limited due to insufficient proliferation [5]. Therefore immortalized proliferating cell lines are often used instead, such as the human THP-1 monocytic leukemia cell line. This cell line is a well-established model, for example in toxicology, immunology and atherosclerosis research, regarding monocyte and macrophage function and biology [6-8].

Phorbol esters, such as phorbol 12-myristate 13-acetate (PMA), are frequently used to elicit the differentiation of THP-1 monocytes into macrophage-like cells which mimic many characteristic features of human primary macrophages [6,9]. The process of differentiation is accompanied by profound changes within the cells, as the cells become adherent and adjust their morphology and physiology [10-12].

Quantitative real-time RT-PCR (RT-qPCR) is a powerful tool for quantifying RNA expression and determining differences in expression levels. Yet, in order to correctly assess the results obtained, a reliable reference is strictly required. As RT-qPCR expression data depend on a multitude of factors, such as amount and quality of isolated RNA, efficiencies of enzymes (reverse transcriptase and DNA-dependent DNA polymerase), and overall variability in transcriptional activity between samples [13-15], it is common procedure to normalize expression of a gene of interest using an internal standard, i.e. a reference gene. Usually genes known to be stably expressed in general, such as GAPDH or ACTB, are chosen as internal standard. Yet the validity of this assumption is rarely verified, although over the past years evidence has emerged that genes previously thought to be stably expressed might actually be regulated under certain conditions [16,17]. Therefore the recently published MIQE (*M*inimum *I*nformation for publication of *Q*uantitative real-time PCR *E*xperiments) guidelines propose reference gene validation for all RT-qPCR experiments [18]. In this study we aimed at identifying suitable reference genes during differentiation of human THP-1 monocytes to macrophages. To the best of our knowledge, this is the first study establishing reference genes for the widely used THP-1 cell culture model of monocyte maturation to macrophages.

Selection of reference genes and proper assessment of the stability of selected genes is a circular problem since normalization is required in order to verify the stability of expression of a particular gene. This problem can be circumvented by using several reference genes in combination [13-15]. Based on this approach different Microsoft Excel-based tools have been developed, which allow selection of the most stably expressed genes from a set of expression data of several potential reference genes. For our study we used the GeNorm applet published by Vandesompele *et al*. [15], the BestKeeper applet developed by Pfaffl *et al*. [14], and the NormFinder applet published by Lindbjerg *et al*. [13].

## Results

**Figure 1:**
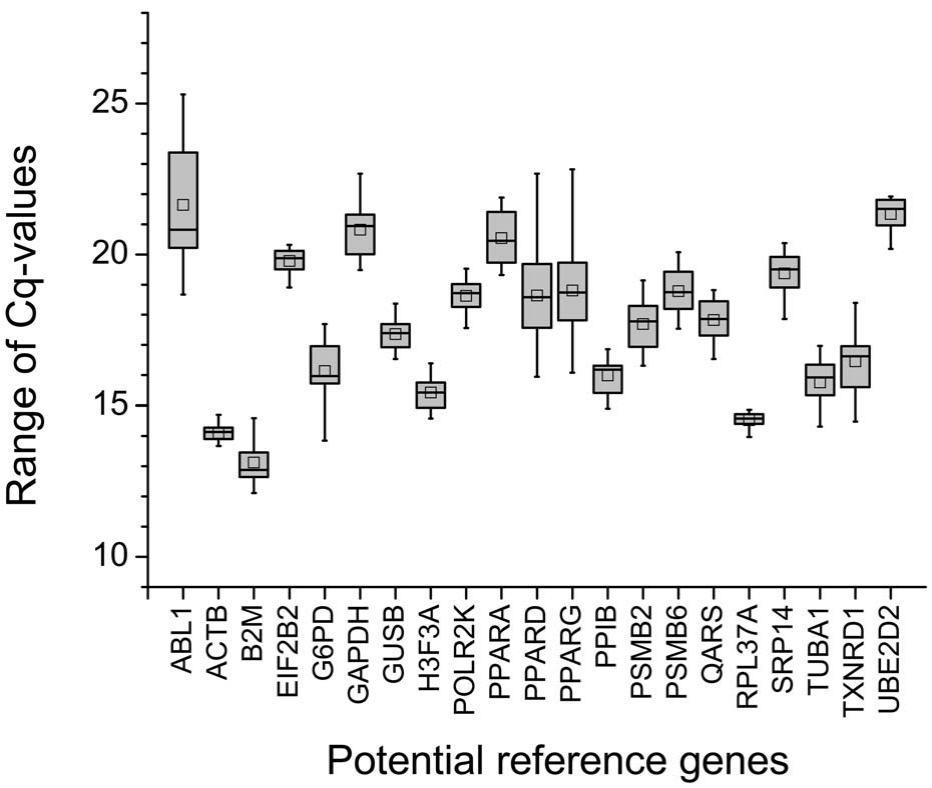
**Observed ranges of C_q _values of the 21 preselected reference gene candidates during phorbol ester-induced monocyte-to-macrophage differentiation of THP-1 cells**. C_q _values were recorded for six days of differentiation as described in the Methods section. Boxes indicate the 25 and 75 percentile, means are indicated by squares, and whiskers represent maximum and minimum data points.

The aim of our study was to identify and validate reliable reference genes for the THP-1 cell culture model of monocyte maturation. For this we have preselected 21 genes from a list of reference genes, which had shown promising little variation in previous experiments [19], as well as from a list of genes often used in studies involving human macrophages [20-23], and thus were considered potential reference genes. Background information on the preselected potential 21 reference genes is provided as Additional file 1, Table S1. The observed range of C_q _values was distributed over a fairly large range representing highly expressed genes (e.g. B2M [C_q _± SD, 13.1 ± 0.7] and ACTB [14.1 ± 0.3]), as well as less abundant mRNAs (e.g. UBE2D2 [21.3 ± 0.6] and GAPDH [20.8 ± 0.8]) (Figure 1). Diagrams representing the variance of C_q _values at each point of measurement for every single gene are available as Additional file 2, Figure S1.

**Figure 2:**
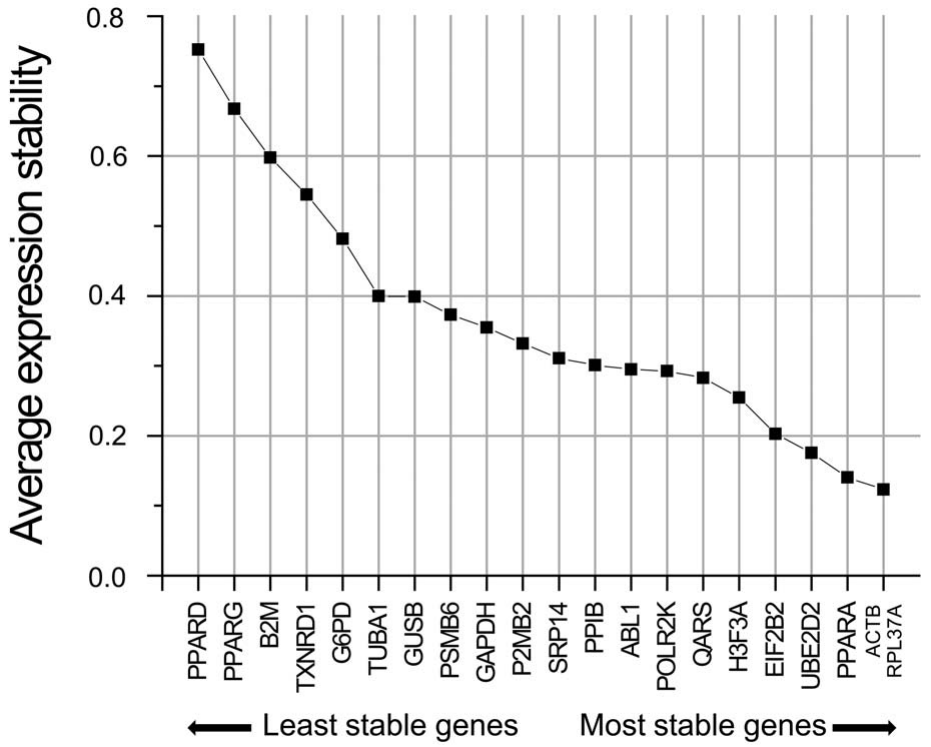
**Average expression stability ranking of the 21 preselected potential reference genes**. Ranking of the potential reference genes as calculated by the Microsoft Excel-based application GeNorm is shown. *ACTB *and *RPL37A *are the most stably expressed genes of the entire set during maturation of THP-1 monocytes into macrophages.

Obtained raw C_q _values were manually transformed into GeNorm data input format and afterwards analyzed by GeNorm. First level analysis performed by GeNorm creates a ranking of all reference genes based on their pairwise variances (Figure 2). This identified the pair of ACTB and RPL37A as the most stable genes of the entire set during differentiation of THP-1 monocytes into macrophages; PPARD and PPARG were the least stable genes (Figure 2), which also show highest variation of C_q _values during maturation of THP-1 cells (Figure 1).

On the second level of analysis the number of reference genes, which need to be included into the assessment in order to provide a reliable normalization, was determined. For this purpose pairwise variances of the normalization factors were calculated when an additional reference gene is included or not. Vandesompele *et al*. suggested a cut-off at 0.15 [15], i.e. if the inclusion of a further reference gene into the calculation of the normalization factors causes a pairwise variance of less than 0.15 as opposed to the exclusion of the respective reference gene, than this gene has no significant contribution to the normalization factor and does not need to be included into the calculation. On the other hand if the variance exceeds 0.15 there is a significant gain in stability and reliability and the respective reference gene should be considered for the calculations. A graphical representation of the calculated pairwise variances is therefore given as Additional file 3, Figure S2. According to these pairwise variances we recommend to include the first two reference genes (ACTB and RPL37A) only into the normalization factor, since none of the pairwise variances actually exceeds the threshold of 0.15.

**Figure 3:**
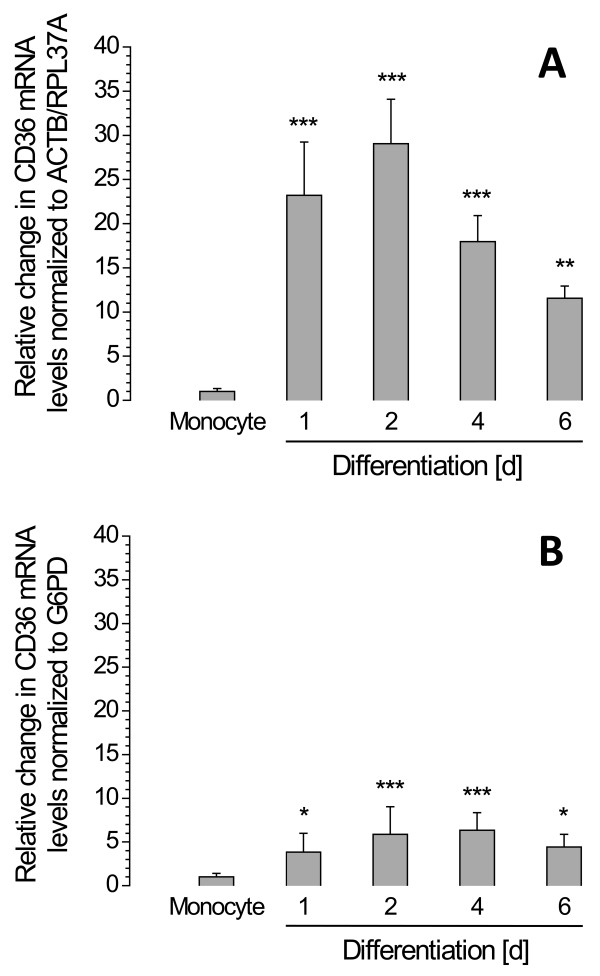
**Comparison of alternative procedures for normalization of CD36 expression data obtained during THP-1 monocyte differentiation**. **A**: Normalization using GeNorm normalization factor (ACTB and RPL37A). **B**: Normalization using G6PD expression data. Different fold changes and statistical interpretations are due to normalization procedures only. Error bars indicate standard deviations. *, *p *< 0.05; **, *p *< 0.01; ***, *p *< 0.001.

In order to demonstrate the importance of combining several reliable reference genes into a set of normalization factors for normalization of measured C_q _values, we show in Figure 3 the results of two different normalizations for a single set of measurements. *CD36*, the gene encoding the scavenger receptor CD36, is known to be regulated during THP-1 monocyte differentiation [24]. The CD36 protein contributes to the uptake of oxidized LDL particles and fatty acids by macrophages [25,26]. The obtained C_q _values were normalized using the two most reliable reference genes identified as described above (ACTB and RPL37A; Figure 3A), and the widely used reference gene G6PD (Figure 3B), respectively. As shown in Figure 3, normalization with G6PD in comparison to ACTB and RPL37A indicates a much less pronounced regulatory effect.

The stability ranking of the reference genes determined by GeNorm was assessed by comparison with two alternative Microsoft Excel-based applets, termed NormFinder [13], and BestKeeper [14]. In Table 1 the rankings produced by these applets are listed next to the GeNorm ranking obtained from our analyses. Interestingly, these rankings differ from each other in the midfield positions yet the top and bottom ranked genes are fairly constant. Especially the genes *ACTB *and *RPL37A *top ranked by GeNorm have been reproducibly listed among the four most reliable genes. For an overview of the ranking calculated by NormFinder see Additional file 4, Figure S3.

**Table 1:** Comparison of stability ranking of the 21 preselected potential reference genes by BestKeeper, GeNorm and NormFinder.

Ranking	BestKeeper	GeNorm	NormFinder
Most stable	RPL37A	RPL37A/ACTB	EIF2B2
	ACTB		GUSB
	EIF2B2	PPARA	ACTB
	GUSB	UBE2D2	RPL37A
	H3F3A	EIF2B2	PPARA
	UBE2D2	H3F3A	UBE2D2
	POLR2K	QARS	SRP14
	PPIB	POLR2K	GAPDH
	SRP14	ABL1	H3F3A
	B2M	PPIB	PSMB2
	QARS	SRP14	PSMB6
	PSMB2	PSMB2	POLR2K
	GAPDH	GAPDH	B2M
	TUBA1	PSMB6	QARS
	PSMB6	GUSB	PPIB
	PPARA	TUBA1	ABL1
	G6PD/TXNRD1	G6PD	G6PD
		TXNRD1	TXNRD1
	PPARG	B2M	TUBA1
	PPARD	PPARG	PPARG
Least stable	ABL1	PPARD	PPARD

## Discussion

Recent studies provide clear evidence that traditional non-validated "housekeeping" genes such as *GAPDH *and *ACTB *are not stably expressed in many experimental setups [27-30], and are therefore not suitable for normalization in a broad range of cell models. Despite this knowledge, the majority of published RT-qPCR studies still lack proper validation of the reference genes. To reduce the risk of erroneous results due to instable reference gene expression we aimed at validating reference genes for the widely used THP-1 cell line which allow reliable RT-qPCR analyses of mRNA expression during maturation of monocytes into macrophages.

Our analyses of 21 preselected potential reference genes stably expressed in a broad range of tissues revealed that during differentiation of THP-1 monocytes to macrophages the normalization using a GeNorm normalization factor calculated from ACTB and RPL37A mRNA expression data is the most stable and reliable option and is sufficient for an accurate assessment of relative changes in gene expression. In contrast to this, other very frequently used reference genes, such as GAPDH and G6PD, are much less reliable. Additionally to the lack of reliability a normalization using G6PD, as shown in Figure 3, yields considerably different results when compared to normalization using the reference genes ACTB and RPL37A as recommended by GeNorm. Thus, the frequently used reference gene G6PD is actually not suitable for normalization and indeed may cause misinterpretations of results.

A comparison with recently published studies involving monocytes and macrophages further highlights the importance of individual validation of reference genes even if comparable cell models are used. For example, Piehler *et al*. validated reference genes in lipopolysaccharide-stimulated primary human monocytes isolated from peripheral blood [31]. In contrast to our results, Piehler and colleagues identified *PPIB *and *B2M *as most stably expressed genes in primary human monocytes, while *ACTB *was found to be inapplicable as reference gene. In our THP-1 monocyte maturation model, *ACTB *is the most stably expressed gene whereas PPIB and B2M are inappropriate reference genes. Furthermore, *ABL1 *und *GUSB*, two reference genes often used in studies involving leukocytes and monocytes [20-22], failed both as reliable reference genes in our hands. Our findings emphasize the need of appropriate and careful validation of reference genes for cell culture models in general and the THP-1 model system in particular.

We also asked the question whether further promising reference gene candidates stably expressed during THP-1 maturation may exist. To answer this question we compared our findings with microarray data on mRNA expression in monocytes and macrophages of different origin that were available in the NCBI GEO database at the time of writing (20^th ^September 2010). Since distinct model systems and experimental settings require individual validation of reference genes, we considered studies only closely resembling our own experimental setup. Three studies using a monocyte-to-macrophage maturation model involving either THP-1 or primary human cells were available (GEO entries GDS3554, GDS3203 and GDS2430) [32-34]. Unfortunately, these microarray raw data online provide average signal intensities for more than 20.000 probes but lack information on the specificity of the signal and whether the respective probe of the corresponding Affymetrix microarray was called present or absent. Thus, a comparative assessment of our selection of reference genes using these microarray data has severe limitations, because many of the low abundant genes detected with the Affymetrix microarrays may represent non-specific background. Nevertheless, we found that *ACTB *was ranked among the genes showing least variances of average signal intensity across all samples, while *RPL37A *was ranked in a midfield position and *G6PD *was among the probes with highest variances of average signal intensity. With the limitations in mind, these findings indicate that our selection of reference genes holds true also on a broader scale. However, further stable reference genes may be available for THP-1 cells in global gene expression data in public databases but further experimental validation is required prior using them for normalization of RT-qPCR data.

Apart from GeNorm other Microsoft Excel-based applications for determination of appropriate reference genes have been published; NormFinder [13] and Bestkeeper [14] were therefore used to assess the quality of the ranking obtained by GeNorm. Comparing the results of the three different applets it is remarkable that the top and the bottom ranked genes more or less retain their ranking independent of the applet used, while some of the genes ranked in the middle positions have changed their ranking quite considerably. The reproducibility of the top rankings gives high confidence regarding the actual stability of the selected reference genes. The lack of confidence within the middle positions might be due to only very slight differences in expression stability within that range (as seen in Figure 2). We therefore conclude that minimal differences within the calculation algorithms of each applet may cause the observed differences in ranking positions.

## Conclusion

To sum up, our study provides clear evidence for the necessity to carefully validate reference genes for normalization of gene expression data obtained by RT-qPCR. As demonstrated by normalizing *CD36 *expression data using validated reference genes (*ACTB *and *RPL37A*) as well as the non-validated reference gene *G6PD*, application of appropriate reference genes may have a significant impact on experimental results. Our study has clearly shown that a GeNorm normalization factor calculated from ACTB and RPL37A mRNA expression is the most stable and reliable option for any RT-qPCR-based expression data obtained during THP-1 monocyte differentiation.

## Methods

### Cell culture

THP-1 monocytes were obtained from ATCC (Manassas, Virginia, USA) and cultured according to the supplier's recommendations. Differentiation of THP-1 monocytes into macrophages was initiated by adding 100 ng/ml phorbol-12-myristate-13-acetate (PMA, Sigma-Aldrich, Seelze, Germany) and 50 μM β-mercaptoethanol (Sigma-Aldrich) to RPMI 1640 medium (PAA, Cölbe, Germany) according to standard protocols [35]. Cells were cultured at 37°C in a humidified 5% (v/v) CO_2 _air atmosphere. Cells were allowed to differentiate for 1, 2, 4 or 6 days, before they were harvested for RNA isolation. A sample of undifferentiated monocytes was included as well.

### RNA isolation

Total RNA was prepared from cell lysates using Qiagen RNeasy Mini kit (Hilden, Germany) according to manufacturer's instructions including an on-column DNase I (Qiagen) digestion as previously reported [36]. Adequate RNA quality was assessed both by agarose gel electrophoresis and photometrically (Additional file 5, Table S2) and was in each case found to be appropriate; all 260/280 ratios were above 2.0. 260/230 ratios were between 1.0 and 2.0. In principle 260/230 ratios should be larger than 2.0 for pure RNA because lower values indicate the presence of contaminations such as guanidine thiocyanate and phenol-chloroform [37]. Since we did not apply phenol extraction absorbance at 230 nm is caused by guanidine thiocyanate only. Previous studies have shown that even very small quantities of guanidine thiocyanate already significantly influence 260/230 ratios, albeit have no measurable effect on downstream applications such as RT-qPCR until concentrations of more than 100 mM [37]. According to Qiagen's technical documentation our samples are suitable for reliable RT-qPCR analyses [37].

### cDNA synthesis

Synthesis of cDNA was performed using Revert Aid First strand cDNA synthesis kit (Fermentas, St. Leon-Rot, Germany). For each cDNA synthesis 5 μg of total RNA and 0.5 μg oligo(dT) primers were used as previously described [38]. Complementary DNA was diluted tenfold and stored at -30°C prior to PCR analyses.

### Real-time RT-PCR

Real-time PCR analyses were performed on a LightCycler 480 II instrument (Roche Diagnostics, Mannheim, Germany) using the Qiagen QuantiTect SYBR Green PCR kit as previously described [38]. Primers were designed using PrimerExpress software version 2.0.0 (Applied Biosystems, Weiterstadt, Germany) and are listed in Table 2. All primer pairs were designed to have melting temperatures of about 60°C and both primers of a primer pair are located in different exons. Primers were purchased from Invitrogen (Karlsruhe, Germany). PCR runs included a 15 min pre-incubation at 95°C to allow heat activation of polymerase, followed by 40 cycles of a two-step PCR consisting of a denaturing phase at 94°C for 15 s and a combined annealing and extension phase at 60°C for 60 s. After completion of PCR a melting curve was recorded. PCR results were analyzed using the LightCycler software release version 1.5.0.39 (Roche Diagnostics). Quality of PCR amplicons was assessed by melting point analysis in order to exclude the formation of primer dimers or other by-products. Size of PCR products was confirmed by agarose gel electrophoresis. The fit point algorithm of the LightCycler software was used to calculate C_q _values. In order to determine expression levels and their changes during maturation of THP-1 cells relative quantifications were calculated using GeNorm normalization factor. In order to calculate the normalization factor raw C_q _values were converted into fold changes, according to the GeNorm manual the fold change of the sample with the highest expression was set to 1. From these values the normalization factor was calculated as the geometric mean of ACTB and RPL37A expression data or G6PD only [15]. After normalization of CD36 data using the calculated GeNorm normalization factor these values were converted into conventional fold changes, by setting the value of the monocyte sample to 1 and scaling all other values proportionally. Samples were prepared in biological triplicates and always measured in technical duplicates.

**Table 2:** PCR primers used in this study.

mRNA	mRNA name	**GenBank accession no**.	**Forward primer**^**1**^	**Reverse primer**^**1**^	Amplicon Size [bp]	PCR efficiency [%]
ABL1	c-abl oncogene 1	NM_005157.3, NM_007313.2	GAGCACAGAGACACCACTGACG	GCTCATCTTCATTCAGGCCG	148	100.9
ACTB	β-actin	NM_001101.3	ATTGCCGACAGGATGCAGAA	GCTGATCCACATCTGCTGGAA	150	97.9
B2M	β-2-microglobulin	NM_004048.2	GCTCCGTGGCCTTAGCTGT	ACGTGAGTAAACCTGAATCTTTGGA	89	101.1
CD36	CD36	NM_001001548.2, NM_001001547.2, NM_000072.3, NM_001127443.1, NM_001127444.1	TCACTGCGACATGATTAATGGTACA	ACGTCGGATTCAAATACAGCATAGAT	126	99.2
EIF2B2	Eukaryotic translation initiation factor 2B2	NM_014239.2	TCCACCCCACTCATCGTCTG	TGGCAGGACTTCTTCAGGAGC	105	101.1
G6PD	Glucose-6-phosphate dehydrogenase	NM_000402.3, NM_001042351.1	CCGTCACCAAGAACATTCACG	GGACAGCCGGTCAGAGCTCT	107	98.7
GAPDH	Glyceraldehyde-3-phosphate dehydrogenase	NM_002046.3	CAACAGCGACACCCACTCCT	CACCCTGTTGCTGTAGCCAAA	115	101.7
GUSB	β-glucuronidase	NM_000181.3	AGCTCATTTGGAATTTTGCCG	GAGTGAAGATCCCCTTTTTATTCCC	81	101.8
H3F3A	H3 histone, family 3A	NM_002107.3	TGGCGCTCCGTGAAATTAGA	TGAGCAATTTCTCGCACCAGA	91	97.0
POLR2K	Polymerase (RNA) II (DNA-directed) polypeptide K	NM_005034.3	ACCCAGAAGGACGTTCAACCTC	TCTCTGCATCTGATTGGATCCC	107	98.9
PPARA	Peroxisome proliferator-activated receptor α	NM_001001928.2, NM_005036.4	AGCCCCTCCTCGGTGACTTAT	GCTTGAGTCGAATCGTTCGC	172	94.6
PPARD	Peroxisome proliferator-activated receptor δ	NM_006238.3, NM_177435.2	AGAACCGCAACAAGTGCCAG	GCATCCGACCAAAACGGA	87	99.1
PPARG	Peroxisome proliferator-activated receptor γ	NM_138712.3, NM_015869.4, NM_138711.3, NM_005037.5	TTCAGAAATGCCTTGCAGTGG	AGCTTCTCCTTCTCGGCCTG	79	100.6
PPIB	Peptidylprolyl isomerase B (cyclophilin B)	NM_000942.4	ATGGCAAGCATGTGGTGTTTG	CCCGGCTGTCTGTCTTGGT	84	96.8
PSMB2	Proteasome subunit, β type 2	NM_002794.3	ACGGCAGCAGCTAACTTCACA	TGGCCCTTCATGCTCATCA	108	101.9
PSMB6	Proteasome subunit, β type 6	NM_002798.1	GGAATCATCATCGCAGGCTG	CTGCCTTACCATCATACCCCC	81	101.6
QARS	Glutaminyl-tRNA synthetase	NM_005051.1	GAGCGTCTTGGATATTTCTCCGT	GCTTCCAGCTCACACCTTTCC	108	98.3
RPL37A	Ribosomal protein L37a	NM_000998.4	ATTGAAATCAGCCAGCACGC	AGGAACCACAGTGCCAGATCC	94	97.6
SRP14	Signal recognition particle 14 kDa	NM_003134.4	AGCACTGTGGTGAGCTCCAAG	TCAGCCCATCCATGTTAGCTCTA	82	95.1
TUBA1	α1-tubulin	NM_006009.2, NM_006082.2, NM_032704.3	GCACTACACCATTGGCAAGGA	AACCAGTTCCCCCACCAAAG	122	98.4
TXNRD1	Thioredoxin reductase 1	NM_003330.2, NM_182742.1, NM_182729.1, NM_182743.1, NM_001093771.1	CACAATTGGAATCCACCCTGTC	GCTTGCCCCAGAGCGC	73	99.2
UBE2D2	Ubiquitin-conjugating enzyme E2D 2	NM_003339.2	CCAGATGATCCTTTAGTGCCTGAG	ACATCGCATACTTCTGAGTCCATTC	100	102.5

^1^In each case, forward and reverse primers are located in different exons.

### Statistics

Statistical analyses were performed using Microsoft Excel 2007 and three different Microsoft Excel-based applets: GeNorm, BestKeeper and NormFinder. Applets were used according to the instructions provided by the respective suppliers; this included a transformation of the raw C_q _values into required data input formats for the applets GeNorm [15], and NormFinder [13]. BestKeeper [14] analyses were based on raw C_q _values without transformation. In order to test for statistical significance a one-factor ANOVA with a post-hoc Dunnett test was performed; this test is known to be rather robust against a violation of the normality condition. Statistical significances indicated by the Dunnett test were confirmed by a non-parametric permutation assay.

## Authors' contributions

MBM and SS designed and performed the experiments, analyzed the data and drafted the manuscript. SL designed the study, was involved with the analysis and interpretation of data and critically helped to draft the manuscript. All authors read and approved the final manuscript.

## Supplementary Material

Additional file 1 **Table S1. Gene product function and GO annotation of the genes used as potential reference genes and CD36**. Information was obtained from the NCBI resource http://www.ncbi.nlm.nih.gov/ and the Gene Ontology website http://www.geneontology.org/. Click here for file

Additional file 2 **Figure S1. Summary of observed variances of C_q _values of the 21 preselected potential reference genes**. For each potential reference gene the observed variances of C_q _values at each day of differentiation of THP-1 monocytes to macrophages are shown. Squares indicate mean values. Bars represent standard deviations. Click here for file

Additional file 3 **Figure S2. Determination of the number of genes required for calculating GeNorm normalization factor**. Variances of pairwise combined normalization factors were calculated in order to determine which genes had to be considered for inclusion into the GeNorm normalization factor. Each bar represents the variance of the normalization factors when an additional gene is included into the calculation; the starting set of normalization factors is calculated from the two most stable genes (*ACTB *and *RPL37A*). Further genes are included according to the stability ranking calculated previously; according to Vandesompele *et al*. further genes are recommended to be included until the variance is below 0.15 [1]. **Reference **[1] Vandesompele J, De Preter K, Pattyn F, Poppe B, Van Roy N, De Paepe A, Speleman F: **Accurate normalization of real-time quantitative RT-PCR data by geometric averaging of multiple internal control genes**. *Genome Biol *2002, **3**:research0034.1-0034.11. Click here for file

Additional file 4 **Figure S3. NormFinder analysis showing logarithmic intergroup and intragroup variances of the 21 preselected reference genes and CD36**. NormFinder application was used to calculate inter- and intragroup variances as an estimate of gene stability [2]. Squares indicate intergroup variance. Bars represent intragroup variance. Two distinct groups were defined: Group 1 is constituted of expression data measured for undifferentiated THP-1 monocytes; group 2 combines all expression data of differentiating and differentiated THP-1 macrophages. A gene's stability is represented by the distance of the respective square from the horizontal line at 0. **Reference **[2] Lindbjerg CA, Jensen JL, Ørntoft TF: **Normalization of real-time quantitative reverse transcription-PCR data: A model-based variance estimation approach to identify genes suited for normalization, applied to bladder and colon cancer data sets**. *Cancer Res *2004, **64**:5245-5250. Click here for file

Additional file 5 **Table S2. 260/280 and 260/230 ratios for assessment of RNA quality**. RNA quality was assessed photometrically using an Eppendorf BioPhotometer plus. Click here for file
