# Supplementary material for: Selection of reliable reference genes during THP-1 monocyte differentiation into macrophages
Source: BMC Mol Biol. 2010 Dec 1;11:90. doi: 10.1186/1471-2199-11-90 (PMC3002353; doi:10.1186/1471-2199-11-90)
Supplement: Additional file 1 — Table S1. Gene product function and GO annotation of the genes used as potential reference genes and CD36. Information was obtained from the NCBI resource http://www.ncbi.nlm.nih.gov/ and the Gene Ontology website http://www.geneontology.org/. [file 1471-2199-11-90-S1.PDF]

Table S1: Gene product function and GO annotation of the genes used as potential reference genes and CD36.

Gene	mRNA name	Gene product function	GO annotation
ABL1	c-abl oncogene 1	Receptor tyrosine kinase	Signal transduction, cytoskeleton
ACTB	β-actin	Protein of the filaments of the cytoskeleton	Cellular component movement, cortical cytoskeleton
B2M	β-2-microglobulin	Serum protein found in association with MHC class I	Antigen processing and presentation of exogenous protein antigen via MHC class 1b, TAP-dependent
EIF2B2	Eukaryotic translation initiation factor 2B2	Essential GTP exchange protein in protein synthesis	Translational initiation
G6PD	Glucose-6-phosphate dehydrogenase	Enzyme of pentose phosphate pathway (nucleic acid synthesis; NADPH production)	Carbohydrate metabolic process
GAPDH	Glyceraldehyde-3-phosphate dehydrogenase	Enzyme of glycolysis	Glycolysis
GUSB	β-glucuronidase	Enzyme of glycosaminoglycan degradation	Glycosaminoglycan catabolism
H3F3A	H3 histone, family 3A	DNA binding protein in nucleosomes	Nucleosome assembly, DNA binding
POLR2K	Polymerase (RNA) II (DNA-directed) polypeptide K	Subunit of eukaryotic DNA directed RNA polymerases	DNA directed RNA polymerase
PPARA	Peroxisome proliferator-activated receptor α	Nuclear receptor binding hypolipidemic drugs and fatty acids	Ligand-dependent nuclear receptor
PPARD	Peroxisome proliferator-activated receptor δ	Nuclear receptor, potent inhibitor of transcription activity of PPARA and PPARG	Ligand-dependent nuclear receptor
PPARG	Peroxisome proliferator-activated receptor γ	Nuclear receptor forming heterodimers with RXRs (retinoid X receptors)	Ligand-dependent nuclear receptor
PPIB	Peptidylprolyl isomerase B (cyclophilin B)	Cyclosporine binding protein, associated with secretory pathway	Protein folding, peptidyl-prolyl cis-trans isomerase
PSMB2	Proteasome subunit, β type 2	Subunit of 20S core of 26S proteasome (multicatalytic proteinase complex)	Proteasome complex (subunit of 20S core of 26S proteasome)
PSMB6	Proteasome subunit, β type 6	Subunit of 20S core of 26S proteasome (multicatalytic proteinase complex)	Proteasome complex (subunit of 20S core of 26S proteasome)
QARS	Glutaminyl-tRNA synthetase	Enzyme linking the amino acid gln with its respective tRNA for protein synthesis	Glutaminyl-tRNA aminoacylation
RPL37A	Ribosomal protein L37a	Protein of 60S subunit of eukaryotic ribosome	Ribosome (60S subunit)
SRP14	Signal recognition particle 14 kDa	Homologous Alu RNA binding protein	Cotranslational protein targeting to membrane
TUBA1	α1-tubulin	Structural component of the microtubule cytoskeleton; forms heterodimers with β-tubulin	Cytoplasmic microtubule
TXNRD1	Thioredoxin reductase 1	Pyridine nucleotide oxidoreductase, reduces thioredoxins, involved in selenium metabolism	Thioredoxin-disulfide reductase
UBE2D2	Ubiquitin-conjugating enzyme E2D 2	Necessary protein for targeting proteins for degradation	Ubiquitin-protein ligase
CD36	CD36	Scavenger receptor	Antigen, cell adhesion, transport
