# Supplementary figures and images for: Selection of reliable reference genes during THP-1 monocyte differentiation into macrophages
Source: BMC Mol Biol. 2010 Dec 1;11:90. doi: 10.1186/1471-2199-11-90 (PMC3002353; doi:10.1186/1471-2199-11-90)

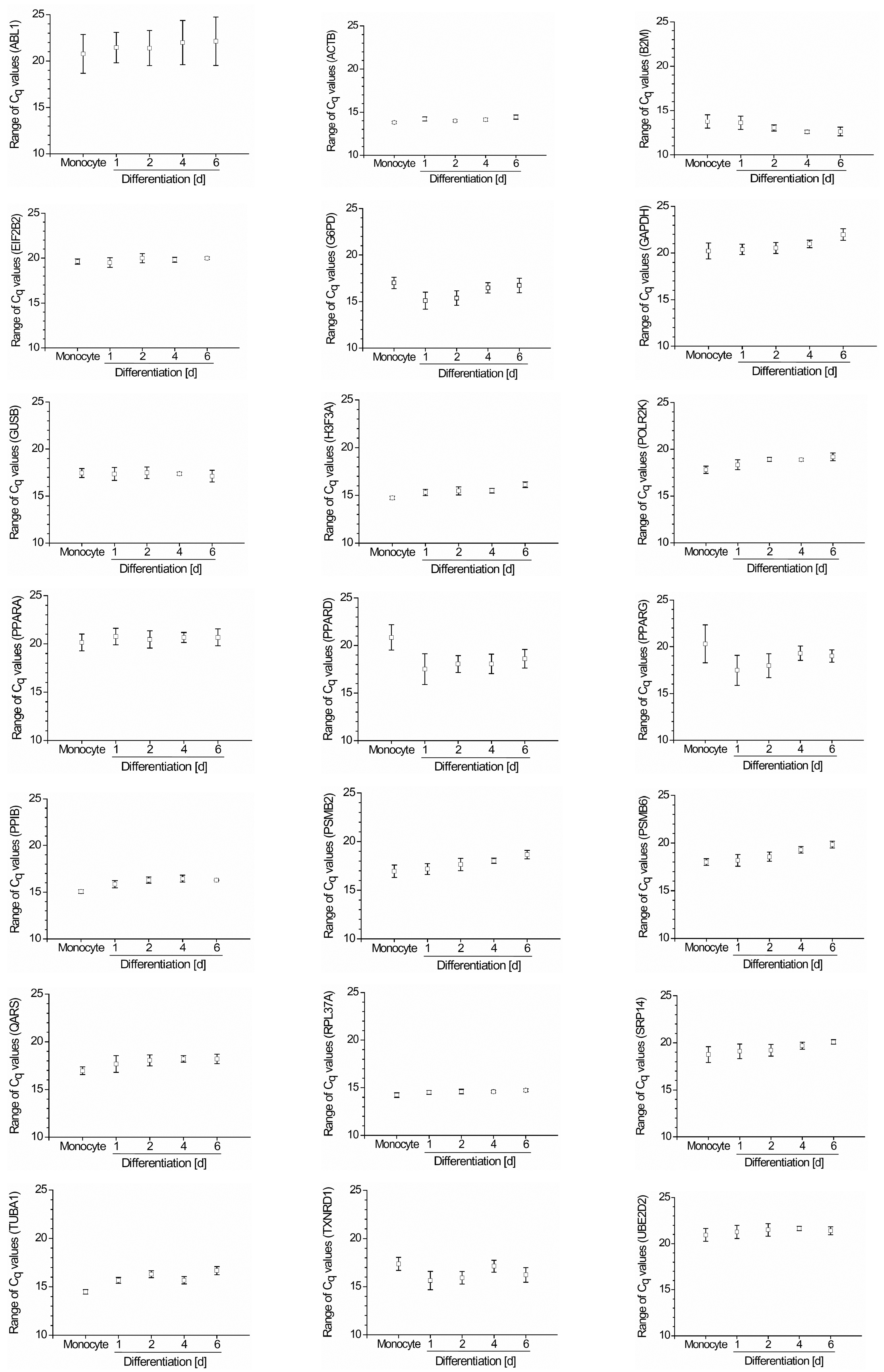

Supplement: Additional file 2 — Figure S1. Summary of observed variances of Cq values of the 21 preselected potential reference genes. For each potential reference gene the observed variances of Cq values at each day of differentiation of THP-1 monocytes to macrophages are shown. Squares indicate mean values. Bars represent standard deviations. [file 1471-2199-11-90-S2.TIFF]

Variance (arbitrary units)

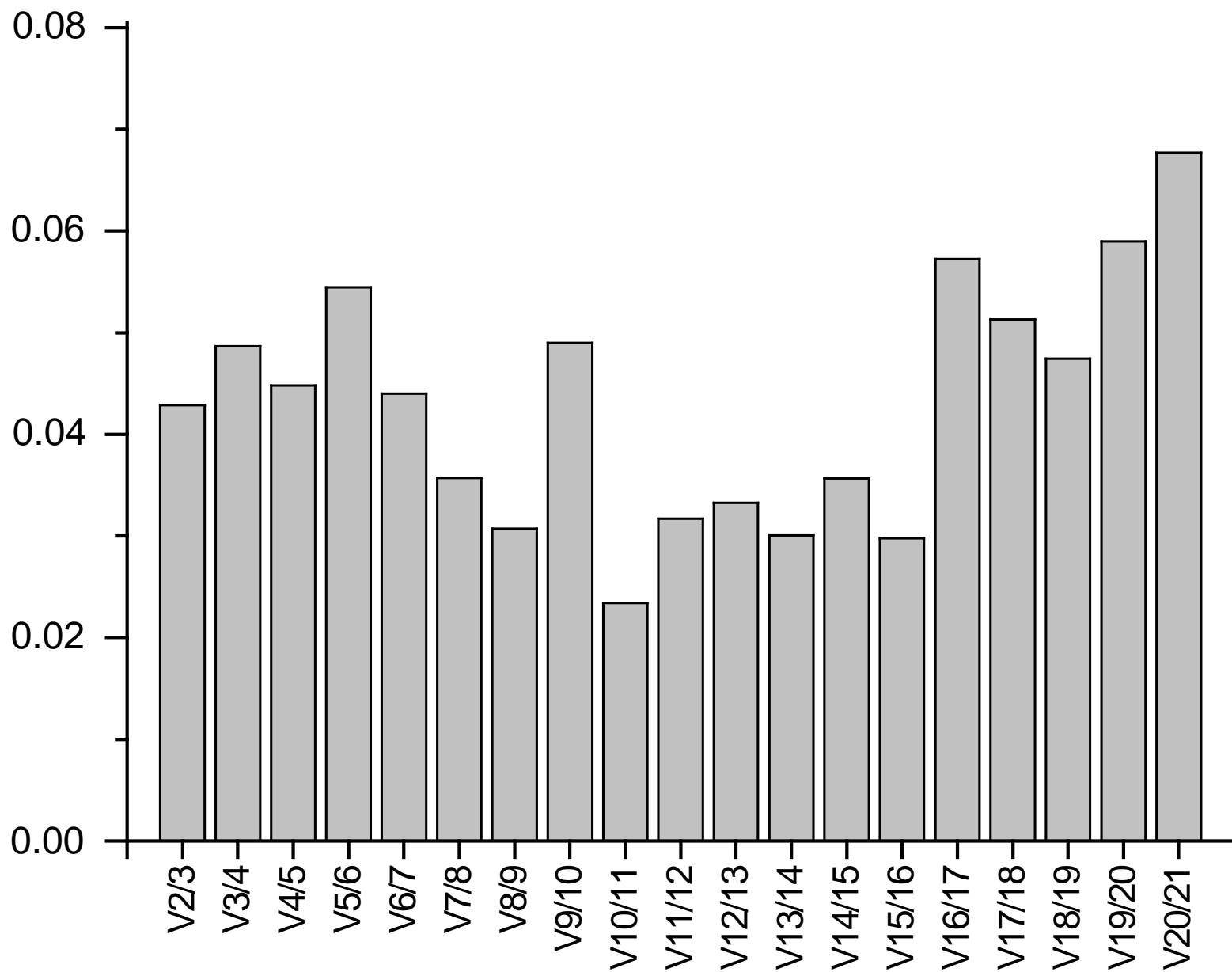

Pairwise combination of normalization factors

Supplement: Additional file 3 — Figure S2. Determination of the number of genes required for calculating GeNorm normalization factor. Variances of pairwise combined normalization factors were calculated in order to determine which genes had to be considered for inclusion into the GeNorm normalization factor. Each bar represents the variance of the normalization factors when an additional gene is included into the calculation; the starting set of normalization factors is calculated from the two most stable genes (ACTB and RPL37A). Further genes are included according to the stability ranking calculated previously; according to Vandesompele et al. further genes are recommended to be included until the variance is below 0.15 [1]. Reference [1] Vandesompele J, De Preter K, Pattyn F, Poppe B, Van Roy N, De Paepe A, Speleman F: Accurate normalization of real-time quantitative RT-PCR data by geometric averaging of multiple internal control genes. Genome Biol 2002, 3:research0034.1-0034.11. [file 1471-2199-11-90-S3.PDF]

log differences between groups

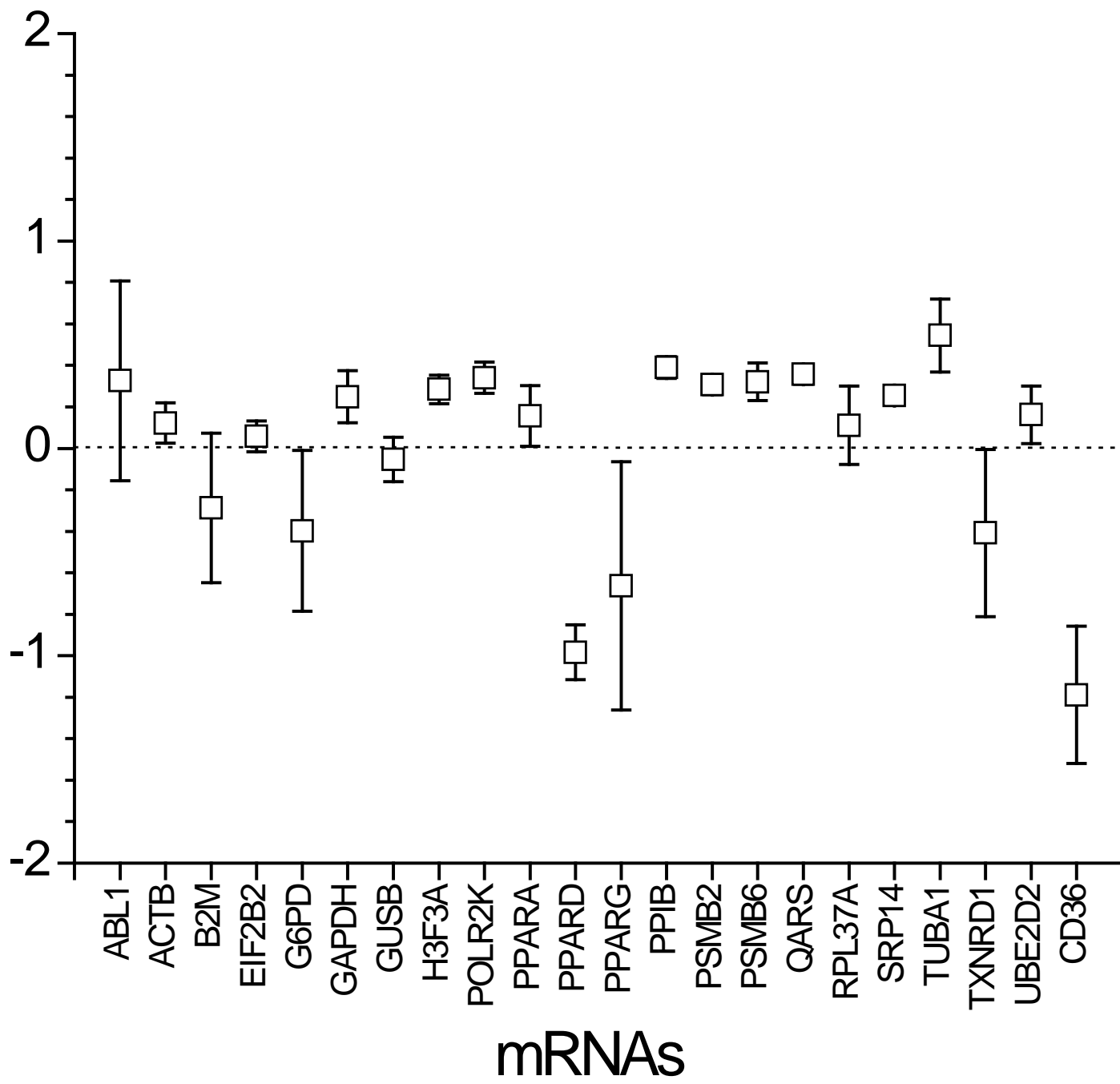

Supplement: Additional file 4 — Figure S3. NormFinder analysis showing logarithmic intergroup and intragroup variances of the 21 preselected reference genes and CD36. NormFinder application was used to calculate inter- and intragroup variances as an estimate of gene stability [2]. Squares indicate intergroup variance. Bars represent intragroup variance. Two distinct groups were defined: Group 1 is constituted of expression data measured for undifferentiated THP-1 monocytes; group 2 combines all expression data of differentiating and differentiated THP-1 macrophages. A gene's stability is represented by the distance of the respective square from the horizontal line at 0. Reference [2] Lindbjerg CA, Jensen JL, Ørntoft TF: Normalization of real-time quantitative reverse transcription-PCR data: A model-based variance estimation approach to identify genes suited for normalization, applied to bladder and colon cancer data sets. Cancer Res 2004, 64:5245-5250. [file 1471-2199-11-90-S4.PDF]
