# Supplementary material for: Selection of reliable reference genes during THP-1 monocyte differentiation into macrophages
Source: BMC Mol Biol. 2010 Dec 1;11:90. doi: 10.1186/1471-2199-11-90 (PMC3002353; doi:10.1186/1471-2199-11-90)
Supplement: Additional file 5 — Table S2. 260/280 and 260/230 ratios for assessment of RNA quality. RNA quality was assessed photometrically using an Eppendorf BioPhotometer plus. [file 1471-2199-11-90-S5.PDF]

Table S2: 260/280 and 260/230 ratios for assessment of RNA quality.

		260/230			260/280		
		**Series A**	**Series B**	**Series C**	**Series A**	**Series B**	**Series C**
Monocytes		1.06	1.85	2.03	2.07	2.08	2.08
Macrophages [Days of differentiation]	1	1.66	1.60	1.72	2.08	2.09	2.09
	2	1.60	1.91	1.43	2.07	2.09	2.09
	4	1.59	1.96	1.83	2.07	2.06	2.05
	6	1.81	1.93	1.87	2.09	2.09	2.05
